# Sport Prosumer Networks: Capital and Value of American Sports During Covid-19

**DOI:** 10.1177/00027642221118293

**Published:** 2022-08-29

**Authors:** Alexander J. Bond

**Affiliations:** 1 Leeds Beckett University, Leeds, West Yorkshire, UK

**Keywords:** prosumption, sport, social network analysis, Twitter, Covid-19

## Abstract

Prosumption capital is underexplored within social media sites, especially within sports. This article explores how the Covid-19 disruptions were used to extract prosumption capital from Twitter. Adopting an economic sociology perspective to measure prosumption capital, 2.3 million tweets were analyzed across the National Basketball Association, Major League Baseball, National Hockey League, and Major League Soccer sports properties. This article applies social network analysis measures, indegree, domain, and proximity prestige to measure prosumption capital and shows how media organizations and other public figures capitalized on the Covid-19 disruptions. It also shows how the structure and those capitalizing through prosumption on Twitter are similar across the sports properties.

## Introduction

During the Covid-19 pandemic, every facet of life was altered across the globe, especially sport and leisure (Parnell, Bond, et al., 2020; Parnell, Widdop, et al., 2020). While disruptions to the production of professional sports in North America are not unique to the Covid-19 pandemic—with occasional work stoppages due to lockouts (owners refuse production) or strikes (players refuse production)—it presented a unique time when four of the five main sports were suspended at the same time. In 2020, the NBA was suspended between March 12th to July 30th (NBA, 2020), the NHL was suspended between March 12th and July 30th (NHL, 2020), the MLS was suspended between March 12th and July 8th (MLS, 2020), and MLB was suspended between March 20th and July 23rd (MLB, 2020). While fans could not consume their teams’ core product, they found a similar utility from engaging in virtual spaces together (Mastromartino et al., 2020; Naraine, 2019). This virtual space is a networked community, relying on prosumption processes, which creates an important structure of communication (Bond et al., 2020, 2021). Furthermore, network theory tells us that position in network structure can offer an advantage (Burt, 1992, 2004; Granovetter, 1985) and thus value (Bond et al., 2020, 2021). However, this prosumed and networked virtual space can also offer utility to other organizations from what Neale (1964) calls the forth-estate benefitting from the production of sport’s core product, like the media. This article offers further empirical insight into sports fans’ prosumption throughout the Covid-19 suspension, partly filling the gap in research applying prosumption principles to sport (Andrews & Ritzer, 2018).

## Relevant Literature

### Prosumption

**Figure 1.**
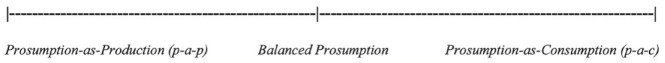
The prosumption continuum (Ritzer, 2015b).

Prosumption identifies the interlinked production and consumption processes, which are mutually interdependent and cannot be separated (Andrews & Ritzer, 2018). While Toffler (1980) introduced the “rise of the prosumer,” it became popular after the 2007 global recession (Ritzer & Jurgenson, 2010; Ritzer et al., 2012). Quintessentially, prosumption has always formed part of everyday life, meandering through different “waves” of society from hunter-gather to postindustrial periodization (Toffler, 1980). As society develops, so does the level of prosumption, which Ritzer (2015a, 2015b) describes as the “prosumption continuum” (Figure 1), identifying all production and consumption rely on elements of the other, just in varying amounts. This provides a scale of prosumption anchored by “prosumption-as-production” and “prosumption-as-consumption,” representing the prosumption required in traditional production and consumption processes. Balanced prosumption depicts those acts which are “more or less evenly balanced” (Ritzer, 2015a, p. 2).

### Prosumption, Digital Leisure, and Web 2.0

As the virtual world develops through technological advancements, the lines between physical and virtual worlds become increasingly blurred, especially through the development of Web 2.0 applications (Castells, 2010, 2015; Ritzer et al., 2012). While acknowledging the ambiguity defining Web 2.0 applications (Constantinides & Fountain, 2007; O’Reilly, 2007; Orenga-Roglá & Chalmeta, 2016), it is clear they are based on prosumption principles, relying on a user-generated production function as users become “active contributors” (Bond et al., 2020, 2021; Lai & To, 2015). This active contribution means the user becomes both producer and consumer. For example, standard users of social media platforms are balanced prosumers since a user *produces* content often by *consuming* another user’s content, which other users *consume*. Their *consumption* often simultaneously *produces* content for others to further *consume*; therefore impossible to distinguish the user as producer or consumer. This cycle continues throughout users’ direct and indirect connections within the platform. Consequently, prosumption in the virtual world is a networked activity that relies on users consuming and producing digital leisure opportunities for others, especially during Covid-19 (Bond et al., 2020, 2021; Lashua et al., 2021).

## Prosumer Networks, Capital, and Value

Economic sociology is useful for understanding networked prosumption, offering explicit theories and perspectives, as well as a methodology (Bond, 2021; Wellman, 1988). In particular, embeddedness theory emphasizes how social networks are at the heart of economic activity, specifically how structure and position influence behavior (Granovetter, 1985, 2017; Knoke, 2012). Bond et al. (2021) argue how this structural position creates value through prosumer capitalism. Ritzer (2015b) identifies how prosumption capitalists profit from a “definite quantity of other people’s unpaid labor” (Marx, 1867 [2001], p. 534). Such a claim becomes even more true for digital prosumption, whereby sports fans’ digital leisure engagement produces content for others’ consumption (Fuchs, 2014). This prosumption capitalism extends beyond the Web 2.0 application itself, which directly profits from user’s unpaid labor. Organizations that rely on sports property like media, broadcasters, bookmakers, and sponsors, among others, to produce their own product (see Neale, 1964), can also capitalize on this prosumption system and can capitalize on the digital leisure of sports fans.

Prosumption capitalism within the virtual world can be explored through Granovetter’s (1985) embeddedness theory. Specifically, this article has two elements: overall network structure and individual advantageous position. The overall network structure influences the behavior and microtransactions within the network, reflecting back on the overall network structure. The more cohesive a network or subcommunity is, the more embedded ideas, values, beliefs, and behaviors are among people. The first question for this article is as follows;

*RQ1: How were the NBA, MLB, NHL and MLS prosumer networks structured throughout Covid-19 disruptions?*

On an individual level, Burt (1992, 2004) identified how position within the overall structure could be advantageous, mainly through brokering relationships/connections within the network structure. Here, individuals extract value by being key and influential to the flow of information throughout the network. These users can be considered prosumer capitalists, since their position is created by the prosumption of others, not necessarily their endeavors. Thus, the second and third questions for this article are as follows,

*RQ2: Who are the influential users within the NBA, MLB, NHL and MLS prosumer networks throughout Covid-19 disruptions?*
RQ3: What factors lead to increased influence within the NBA, MLB, NHL and MLS prosumer networks throughout Covid-19 disruptions?

## Methodology

### Data

To measure digital prosumption capital during Covis-19 suspension, data was scraped from web 2.0 application Twitter. The search term was kept simple to NBA, MLS, NHL, and MLB using the time frame from suspension to resumption for each sports property, as shown in Table 1, to provide a nominalist boundary for analysis (Borgatti & Halgin, 2011; Knoke & Yang, 2008). All conversation data, including tweets, mentions, likes, retweets, and quoted tweets, were collected using the rtweet package (Kearney, 2019), academictwitteR package (Barrie & Ho, 2021), and the tidyverse package (Wickham, 2019) all within R Studio programming software (R Core Team, 2020). To respect Twitter’s Application Programming Interface (API) and the conditions of fair usage of Twitter’s academic research license, the total tweets collected were limited to roughly 1 million.

**Table 1.** Brief Overview of Data Collection.

Search Term	Dates scraped	Scraped tweets	Cleaned tweets	Collected users
“MLB”	March 20th to July 23rd	1,000,344	657,672	392,102
“NBA”	March 11th to July 30th	1,000,296	635,445	430,659
“NHL”	March 12th to July 30th	1,001,750	659,037	328,984
“MLS”	March 12th to July 8th	1,000,286	366,880	284,872

Data were analyzed using the network package (Butts, 2008) also in the R Studio environment and visualized using Gephi software (Bastian et al., 2009). Once data was collected, a sample was qualitatively checked to ensure all tweets related to the respective sports property; none were removed because of relevance. Since this article focuses on the dyadic relationship of users, any isolated tweets were removed. Table 1 demonstrates the final data set used in this article.

### Social Network Analysis

Since this article presents Twitter as a network of prosumption and is interested in the relationship between users’ dyadic (and triadic) relationship, social network analysis seems the most logical (Bond et al., 2020, 2021). A network (or graph) describes a set of elements, termed vertices (or nodes) connected through interactions, and relationships, termed edges. Following Wasserman and Faust (2009), a graph is noted as *G*(*V,E*) where *V* is a set of vertices and *E* a set of edges connecting vertices, *L* ∈ *V×V* (Borgatti et al., 2018). An edge connecting vertices *x* and *y* in graph *G* would be written (*a,b*) ε *E(G)*. In the context of using Twitter, vertices are users (@user1, @user2, @user3, @user . . . *n*) and edges are the relational tie connecting a user, which can be; “Retweet”—@User1 shares @User2’s content; “RepliesTo”—@User1 creates a message starting with @User2 and finally “Mentions” where @User1 creates a message containing but not starting with @User2. A “Tweet” is a message created by @User1 that does not mention another user, but this can be “ReTweeted” or “Liked” by @User2. Therefore, edges follow a direction from @User(*i*) to @User(*j*), so *L_ij_* ∈ {0,1}, with *L_ij_* = 1 showing a connection (like, mention, retweet, or reply) and *L_ij_* = 0 where a connection does not exist. This can be represented in an asymmetric adjacency matrix, **A** = *n* × *n* (*n* representing the number of nodes in the network). Hence, *L_ij_* in the adjacency matrix **A** is not equal to *L_ji_*. Data were analyzed using the network package (Butts, 2008) and visualized using Gephi software (Bastian et al., 2009).

### Overall Network Structure

To understand the overall structure and answer RQ1, Blondel et al. (2008) modularity algorithm was applied since it performs well with large networks and is relatively less computationally heavy. High modularity shows that the overall structure comprises smaller, densely connected communities (or modules) with sparse connections to other communities. Based on the community structure, Himelboim et al. (2017) proposed different typologies of Twitter topic networks. The common hub-and-spoke star-shaped structure, where only a few nodes take a central position, who are often “experts” (Park & Thelwall, 2008; Wang et al., 2010; Welser et al., 2007). Depending on the direction of the connection, this can be either broadcast (many nodes pointing to one or few) or support (one node pointing to many), the former often representing traditional media processes (Himelboim, et al., 2017).

However, the hub-and-spoke structure is not dense and cohesive, as there is little connection between nodes, and it offers little prosumption capital. Divided or unified provide the most prosumption capital since they both have high density, which leads to embeddedness within the network (Granovetter, 1973; Coleman, 1990). The difference between divided structures is highly clustered; unified is not. Thus, a high modularity score demonstrates a divided structure that provides more embeddedness and prosumption capital.

### Measuring Individual Prosumption Capital

This article proposes three prestige network measures to measure prosumption value at the individual level: indegree prestige, domain prestige, and proximity prestige. Indegree prestige is straightforward and is the number of nodes in the network directly pointing to node *v*. This reflects direct prosumption capital, as it shows who is directly benefitting from sports fan’s prosumption, thus also influential in the network (Newman, 2010). This measure is normalized to between 0 and 1 where 1 would mean all other nodes point to node *v* and 0 means none.

Since embeddedness is also about the connections of connections (Granovetter, 1985), domain prestige is also calculated. This measure reflects the proportion of nodes interacting directly or indirectly with node *v* (Lin, 1976; Wasserman & Faust, 2009). Domain prestige also captures any indirect prosumption capital gained from further prosumption from unconnected nodes to node *v.* This measure is also normalized to between 0 and 1, reflecting the proportion of the network that points toward node *v*, whether directly or indirectly.

To account for Burt’s (1992) position that connecting unconnected nodes provides an advantage, in this case capital, proximity prestige was calculated. Proximity prestige takes the domain prestige for node *v* and divides it by the average geodesic distance of a node’s connections to identify its importance in creating proximity for other nodes (Zhao, 2015). Again, between 0 and 1, the higher proximity prestige shows node *v* has an advantageous position created by the direct prosumption of others.

### Understanding User Characteristics of Prosumption Capital

To understand what user factors lead to increase prosumption capital, each individual prosumption capital measure was modeling for each sports property. To do so, the following basic OLS model was used

$$\gamma =\beta_{user\_verified}+\beta_{uuser\_tweet\_count}+\beta_{user\_followers\_count}+\beta_{user\_following\_count}+\varepsilon$$

where γ is either indegree prestige γ_i_, domain prestige γ_d_, or proximity prestige γ_p_.

β is four user profile variables for user *v*, user_verified is a dummy variable identifying when a user is verified or not, 1 = verified, 0 otherwise. Verified account to Twitter shows “that an account of public interest is authentic . . . [and the] your account must be authentic, notable, and active” (Twitter, 2022). user_tweet_count is the total number of tweets, tweeted by user *v*, user_followers_count is the total number of other users following user *v*, and user_following_count is the total number of other users user *v* follows. ε is the error term.

## Findings

### Overall Network Structure

**Figure 2.**
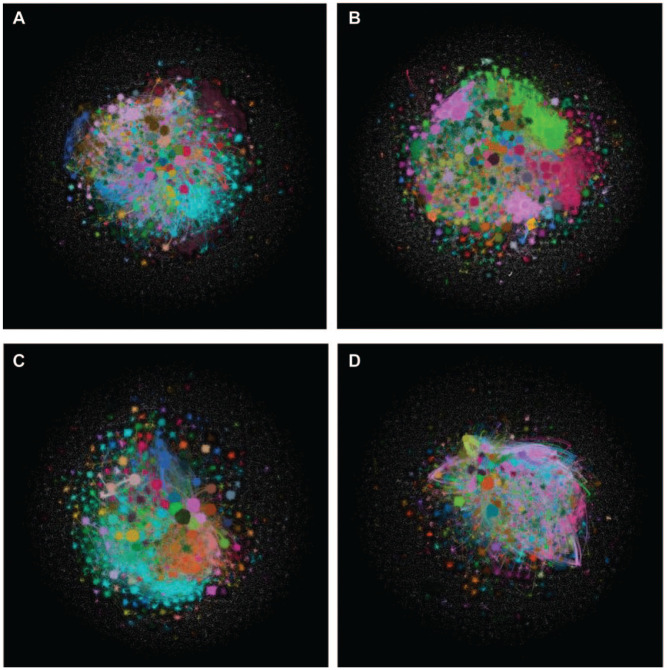
Topological properties of NBA, MLB, NHL, and MLS Twitter networks during Covid-19: (a) MLB, (b) NBA, (c) MLS, and (d) NHL.

**Figure 3.**
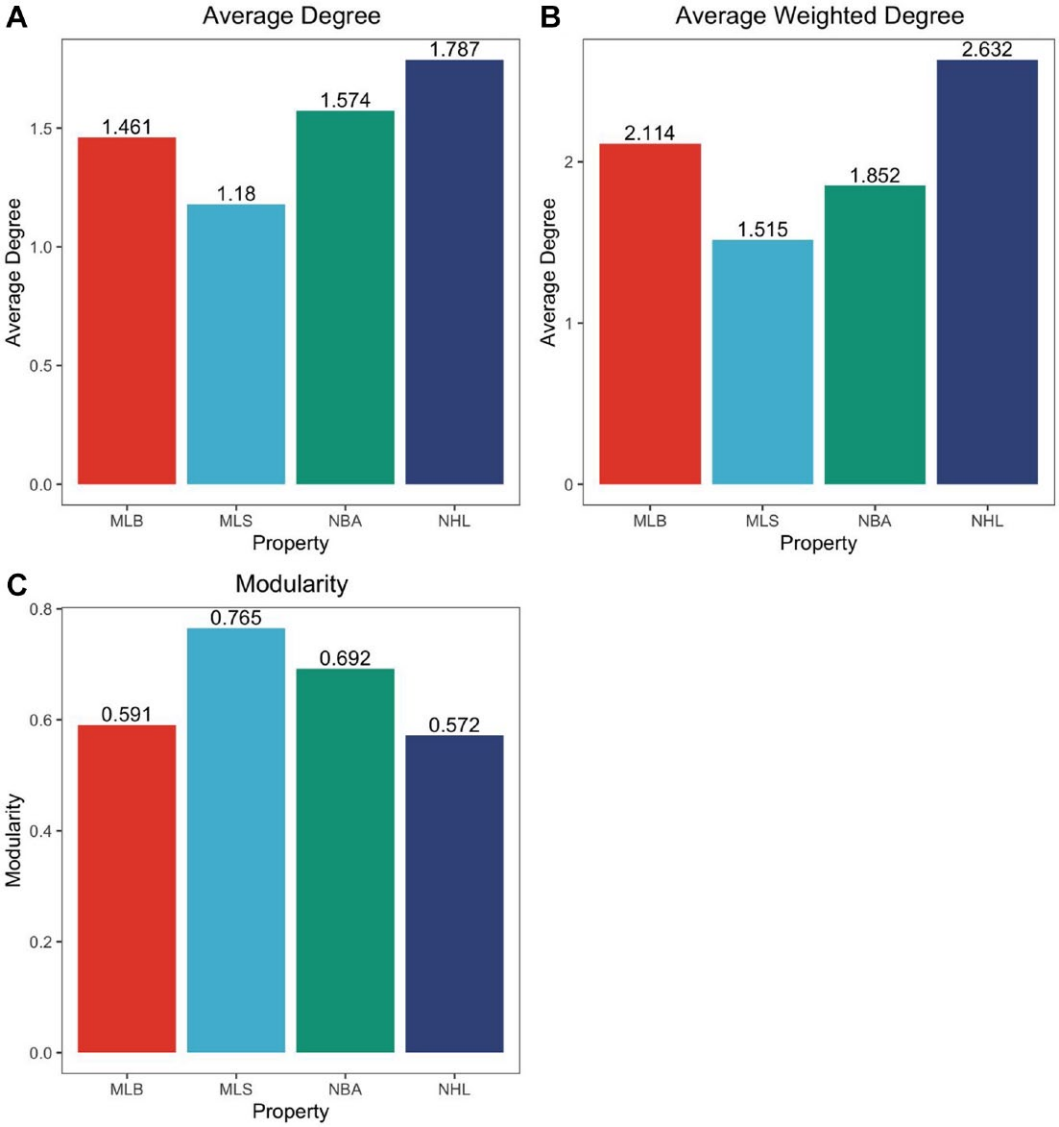
Overall structure of NBA, NHL, MLB, and MLS networks during Covid-19 disruptions: (a) average degree, (b) average weighted degree, and (c) modularity.

The overall Twitter networks for the NBA, NHL, MLB, and MLS during Covid disruptions are visualized in Figure 2(a) to (d). Visual inspections show all follow a similar structure, with a core of heavy prosumption activity and a periphery of minimal prosumption activity, similar to previous Twitter topic research (Bond, 2021). Furthermore, each network has many clusters of activity suggesting a divided network structure with pockets of dense communities (Himelboim, et al., 2017). This is also demonstrated by modularity scores ranging from .57 to .77, which show the overall structure is relatively clustered. The MLS and NBA have higher modularity scores (.77 and .69, respectively), showing users are engaging in prosumption communities more, thus producing more prosumption capital than the MLB and NHL, as shown in Figure 3(c). On average, NHL fans engage in more prosumption, with each user engaging with 1.8 other users, prosuming on average 2.6 times. While the MLS has the least prosumption behavior, the modularity score shows it occurs in a more divided structure, in pockets of densely connected communities, with minimal connection to other communities (Himelboim et al., 2017).

### Individual Prosumption Capital

Tables 2 to 5 show the results of the top 10 users based on indegree, domain, and proximity prestige. These users are all extracting prosumption capital by occupying advantageous positions within the network through the prosumption of other users. Interestingly, the media outlet Bleacher Report (@BleacherReport) and its affiliated accounts (@BR_NBA, @BRGaming, @brfootball, @brhoops, @BRWalkoff, @BROpenIce) dominate both indegree and domain prestige, suggesting they capitalize on both direct and indirect prosumption across all sports properties under investigation. Each sports property’s official account is the most prominent regarding indegree prestige, except for the NBA. Most sports properties benefitted from prosumption capital during covid-19 disruptions, mainly directly. The NBA, on the other hand, created less, with the Bleacher Report NBA account (@BR_NBA) benefitting more. This may reflect a different strategy by @NBA during the Covid-19 disruptions than other sports properties.

**Table 2.** Top 10 NBA Users.

Indegree prestige		Domain prestige		Proximity prestige	
Label	Score	Label	Score	Label	Score
BR_NBA	0.0646	BRGaming	0.4981	NBA	0.1429
NBA	0.0593	brfootball	0.4932	NBA_Africa	0.1169
goknickstape	0.0411	NBA2K	0.4932	NBAHistory	0.1163
Jorda_NBA	0.0410	Ronnie2K	0.4932	spurs	0.1153
BleacherReport	0.0356	brhoops	0.4914	NBAonTNT	0.1141
sometimesigif	0.0302	BR_NBA	0.4911	kylekuzma	0.1123
Ballislife	0.0282	BleacherReport	0.4911	WNBA	0.1122
ESPNNBA	0.0260	LBJamesHarden	0.4747	NBA2KLeague	0.1104
SportsCenter	0.0232	KnicksNationCP	0.4746	NBASummerLeague	0.1093
NBA2K	0.0229	goknickstape	0.4744	nbastats	0.1076

**Table 3.** Top 10 MLB Users.

Indegree prestige		Domain prestige		Proximity prestige	
Label	Score	Label	Score	Label	Score
MLB	0.4192	BRWalkoff	0.7525	MLB	0.4529
BRWalkoff	0.0595	BleacherReport	0.7525	MLBPipeline	0.2919
espn	0.0556	CBSSports	0.7284	jonmorosi	0.2891
SportsCenter	0.0288	Reds	0.7228	darenw	0.2829
Mets	0.0214	BarstoolNate	0.7209	Cut4	0.2826
Phillies	0.0193	MLBPlayersMedia	0.7197	CleGuardians	0.2824
BSmile	0.0160	theScore	0.7177	kenleyjansen74	0.2815
MLBNetwork	0.0133	Pirates	0.7176	MLBStats	0.2815
MLBPipeline	0.0130	YanksMagazine	0.7171	MLBPlayersMedia	0.2812
CBSSports	0.0130	Yankees	0.7170	AnthonyDiComo	0.2807

**Table 4.** Top 10 MLS Users.

Indegree prestige		Domain prestige		Proximity prestige	
Label	Score	Label	Score	Label	Score
MLS	0.3405	BleacherReport	0.5456	MLS	0.3420
BleacherReport	0.0532	brfootball	0.5320	ATLUTD	0.2077
brfootball	0.0444	espn	0.5178	LAFC	0.2060
mvzqz	0.0286	barstoolsports	0.5128	TimbersFC	0.2057
FOXSoccer	0.0231	ActuFoot_	0.5063	AustinFC	0.2049
B24PT	0.0227	SC_ESPN	0.5044	SJEarthquakes	0.2039
espn	0.0226	Football__Tweet	0.4995	dcunited	0.2039
barstoolsports	0.0169	BallStreet	0.4992	Koke6	0.2035
rogbennett	0.0138	DeadlineDayLive	0.4987	NYCFC	0.2033
MLS_Buzz	0.0117	mediotiempo	0.4986	MLSWORKS	0.2033

**Table 5.** Top 10 NHL Users.

Indegree prestige		Domain prestige		Proximity prestige	
Label	Score	Label	Score	Label	Score
NHL	0.3304	johnkrasinski	0.7430	NHL	0.3999
johnkrasinski	0.0627	CBSSports	0.7034	johnkrasinski	0.2889
barstoolsports	0.0452	StephMcMahon	0.6918	PR_NHL	0.2782
StLouisBlues	0.0311	DanyAllstar15	0.6910	StLouisBlues	0.2698
PR_NHL	0.0272	espn	0.6907	MapleLeafs	0.2678
penguins	0.0234	strombone1	0.6905	NHLdotcom	0.2673
MapleLeafs	0.0172	GoldenKnights	0.6904	strombone1	0.2653
CBSSports	0.0168	BleacherReport	0.6902	penguins	0.2648
Sportsnet	0.0143	BR_OpenIce	0.6902	Canes	0.2647
Capitals	0.0137	TripleH	0.6900	Canucks	0.2641

When also accounting for indirect prosumption, media accounts further dominate through domain prestige. Specifically, within the NHL network, public figures like actor John Krasinski (@johnkrasinski) and World Wrestling Entertainment figures Steph McMahon (@StephMcMahon) and Triple H (@TripleH) gain influential positions through both direct and indirect prosumption. However, when further accounting for the average shortest paths, official accounts dominate proximity prestige, suggesting that most other users rely on prosuming official content, further demonstrating how Web 2.0 support prosumption capitalism.

### User Characteristics and Prosumption Capital

Tables 6 to 8 show the results of the regression analysis. Indeed, the objective here is not to predict prosumption capital but to identify key user characteristics that influence it. The direct interpretation of coefficients is not the intention since each prosumption capital measure is normalized to each network. Instead, an understanding of which user characteristics influence prosumption capital is noted. A basic OLS regression was applied, with all model diagnostics suggesting normal assumptions are met. Multicollinearity was checked with Variable Inflation Factor (VIF) scores ranging from 1.009 to 1.049, well below general thresholds (James et al., 2013).

**Table 6.** Indegree Prestige Models.

	Dependent variable: indegree prestige (normalized)			
	NHL	NBA	MLB	MLS
user_verified	0.0002*** (0.000009)	0.0001*** (0.000003)	0.0001*** (0.000001)	0.0002*** (0.000016)
user_tweet_count	0.000003 (0.0000002)	−0.000002 (0.000001)	0.000004 (0.000001)	−0.000004 (0.000002)
user_followers_count	0.000011*** (0.000002)	0.000012*** (0.00003)	0.000010*** (0.000004)	0.000006*** (0.000002)
user_following_count	−0.0000001 (0.000001)	0.000002*** (0.00001)	−0.000001 (0.000001)	0.000001* (0.000001)
Constant	0.000001 (0.000002)	0.000003*** (0.0000005)	−0.0000002 (0.000002)	0.000002 (0.000002)
Observations	248,470	338,869	300,173	175,797
*R* ^2^	0.01	0.06	0.02	0.01
Adjusted *R*^2^	0.01	0.06	0.02	0.01
Residual std. error	0.0007 (*df* = 248,465)	0.0002 (*df* = 338,864)	0.0008 (*df* = 300,168)	0.0008 (*df* = 175,792)
*F* statistic	615.2*** (*df* = 4; 248,465)	5,547.3*** (*df* = 4; 338,864)	1,692.1*** (*df* = 4; 300,168)	585.9*** (*df* = 4; 175,792)

* *p < .1*, ***p < .01.

**Table 7.** Domain Prestige Models.

	Dependent variable: domain prestige			
	NHL	NBA	MLB	MLS
user_verified	0.125*** (0.001092)	0.058*** (0.000497)	0.0821*** (0.000703)	0.063*** (0.000813)
user_tweet_count	0.0000002*** (0.0000002)	0.0000001 (0.00000002)	0.000003*** (0.0000002)	−0.000002*** (0.0000001)
user_followers_count	0.000003***	0.000004*** (0.0000002)	0.000002*** (0.0000001)	0.000004*** (0.0000002)
	(0.0000002)			
user_following_count	−0.0000001 (0.00000004)	0.000001*** (0.0000002)	0.0000002*** (0.00000003)	−0.00000003 (0.00000003)
Constant	0.007622*** (0.000186)	0.002401*** (0.000074)	0.001739*** (0.000102)	0.003173*** (0.000117)
Observations	248,470	338,869	300,173	175,797
*R* ^2^	0.05	0.05	0.05	0.04
Adjusted *R*^2^	0.05	0.05	0.05	0.04
Residual std. error	0.085 (*df* = 248,465)	0.039 (*df* = 338,864)	0.051 (*df* = 300,168)	0.044 (*df* = 175,792)
*F* statistic	3,529.72*** (*df* = 4; 248,465)	3,958.17*** (*df* = 4; 338,864)	3,738.17*** (*df* = 4; 300,168)	1,651.57*** (*df* = 4; 175,792)

***p < .01.

**Table 8.** Proximity Prestige Models.

	Dependent variable: proximity prestige			
	NHL	NBA	MLB	MLS
user_verified	0.023*** (0.00018)	0.007*** (0.00005)	0.013*** (0.00011)	0.017*** (0.00017)
user_tweet_count	0.000003*** (0.000000005)	−0.00000001 (0.000000001)	0.0000018*** (0.000000002)	−0.0000012*** (0.00000002)
user_followers_count	0.0000002*** (0.000000001)	0.0000011*** (0.00000001)	0.0000016*** (0.00000001)	0.0000007*** (0.00000001)
user_following_count	−0.00000145** (0.000000067)	0.00000054*** (0.000000015)	0.00000015*** (0.000000041)	−0.00000051 (0.000000040)
Constant	0.0011*** (0.000031)	0.00024*** (0.0000078)	0.00021*** (0.000015)	0.00038*** (0.000017)
Observations	248,470	338,869	300,173	175,797
*R* ^2^	0.068	0.058	0.054	0.049
Adjusted *R*^2^	0.068	0.058	0.054	0.049
Residual std. error	0.0138 (*df* = 248,465)	0.0041 (*df* = 338,864)	0.0075 (*df* = 300,168)	0.0063 (*df* = 175,792)
*F* statistic	4,540.08*** (*df* = 4; 248,465)	5,244.99*** (*df* = 4; 338,864)	4,335.16*** (*df* = 4; 300,168)	2,257.92*** (*df* = 4; 175,792)

* *p*, **p < .05, ***p < .01.

Across all sports properties and all prosumption capital measures whether a user is verified and the number of followers positively influences prosumption capital throughout the Covid-19 disruptions. Interestingly, however, when considering direct prosumption measured by indegree prestige, a user’s activity (user_tweet_count) has little influence on prosumption capital. Therefore, it is not simply the most active users who extract direct prosumption capital. However, when accounting for the indirect prosumption created through dyadic and triadic relations on Twitter, user activity positively influences domain and proximity prestige for the NHL and MLB networks but negatively influences the MLS network and has no influence on the NBA network. Domain prestige is also positively influenced by a user’s following count within NBA and MLB networks but has no significant influence with NHL and MLS networks. Similarly, a user’s following count positively influences prosumption capital within the NBA and MLB networks but negatively influences NHL and MLS networks, showing potential differences between sports fan preferences in the virtual world.

## Discussion

The overall structures of the NBA, MLS, NHL, and MLB networks during Covid-19 disruptions reflect elements of two typologies presented by Himelboim et al. (2017), overall divided clusters with elements of traditional broadcast hub-and-spoke-type structure dominated by media companies. Consequently, the overall structure relies on multiple users who underpin the core structure and reflect in a traditional one-to-many communication system. However, unlike traditional communication systems, users prosume in divided clusters, supporting the embeddedness of shared ideas and values (Granovetter, 1985, 2017). In turn, this provides central users with prosumption capital since they benefit from the unpaid labor of other users (Ritzer, 2015b). Particularly, and by no surprise, media organizations extract the most prosumption capital from the Covid-19 disruptions, specifically the Bleacher Report media outlet. The Bleacher Report was at the forefront of sports information dissemination during Covid-19 disruptions, using multiple user accounts to capitalize on the prosumption behavior of Twitter.

The results show how users with popular platforms can extract prosumption capital, with media outlets and public figures appearing in the top 10, and sometimes more prominent in the network than official accounts, like the NBA, for instance. The regression evidence supports the use of popular platforms to extract prosumption capital, showing that verified users and users with more followers were the most influential user characteristics in creating prosumer capital. This further supports the notion those with popular platforms often gain advantageous positions (Burt, 1992) and can extract prosumption capital. Within Twitter, these are often significant people, experts, public figures, or organizations (Park & Thelwall, 2008; Wang et al., 2010; Welser et al., 2007).

This article has extended previous work examining Twitter topic conversations by blending prosumption theories with network theories (Bond, 2021a, 2021b) and offers further measures to understand prosumption capital (Ritzer, 2015a). It has provided insight into how media and organizations created and extracted value from a time when the core sporting product was absent, effectively profiting from the unpaid labour of sports fans. Importantly, the results show that those who extract prosumption capital are not necessarily the most engaged users by the number of tweets; instead, prosumption capital is gained through popularity. Although this now becomes a chicken and egg paradox, do users become popular due to prosumption capital or is prosumption capital a result of popularity? A question for future research.

The chicken and egg question highlights a limitation of this article, which it does not consider Granovetter’s (1985) temporal embeddedness, which supposes a historical element to any microtransaction, and the overall structure. Understanding the long-term development of prosumption capital and its relationship with popularity would be a logical step and would provide insight into how popularity within the virtual world develops. Further limitations of the current work are that comparisons are not made to the same points when disruptions were not occurring, which would identify if the structures changed or different users capitalized on the Covid-19 disruptions. Also, only four user characteristics are considered provided by Twitter’s API. Therefore, future research should identify more user characteristics that influence prosumption capital or event content characteristics focusing on Tweet level data.
